# Supplementary material for: Adaptive immunity to human coronaviruses is widespread but low in magnitude
Source: Clin Transl Immunology. 2021 Mar 17;10(3):e1264. doi: 10.1002/cti2.1264 (PMC7968850; doi:10.1002/cti2.1264)
Supplement: Supplementary file 1 [file CTI2-10-e1264-s001.pdf]

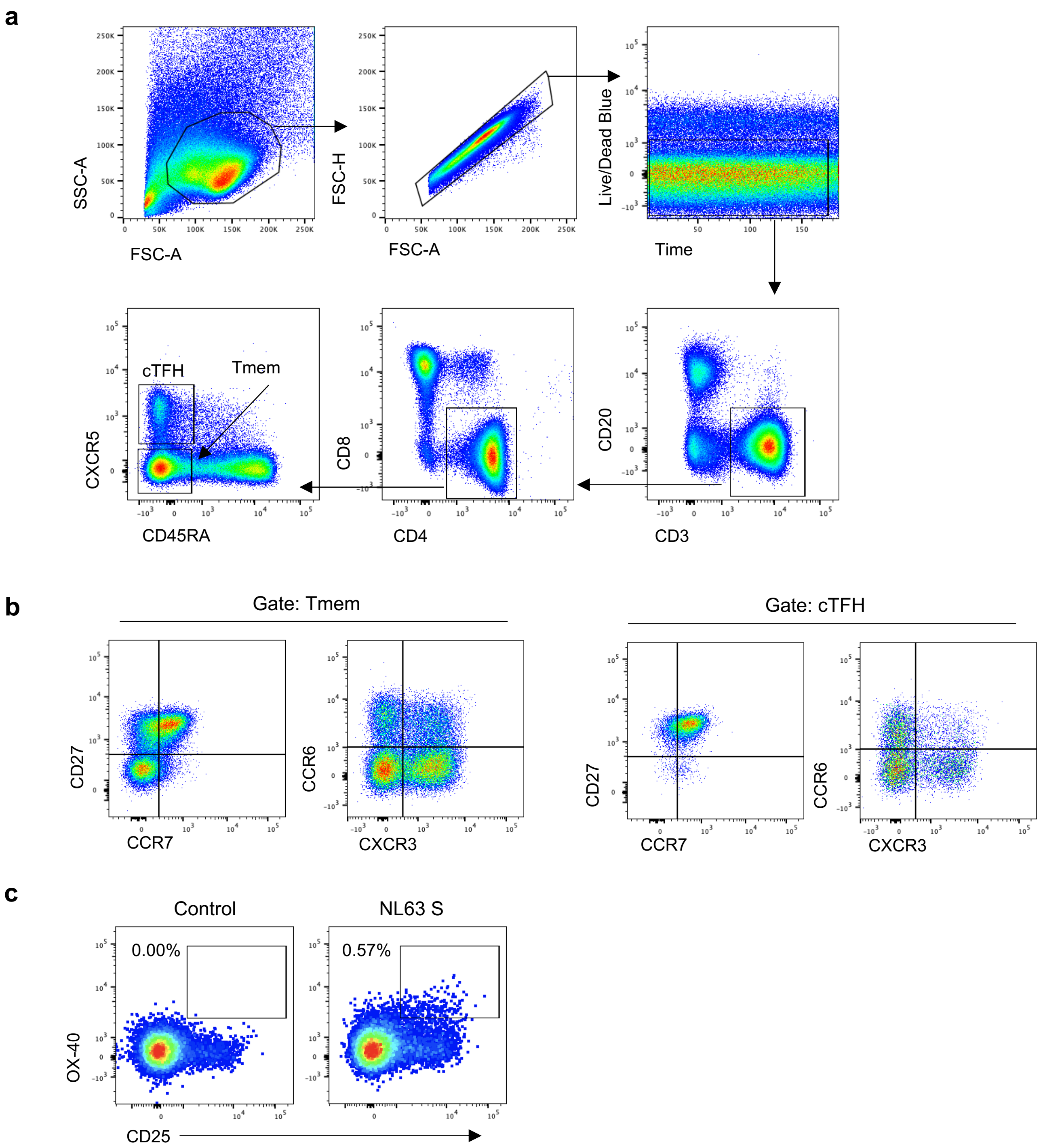

Supplementary figure 1. CD4 T cell gating strategy. (a) Lymphocytes were identified by forward and side scatter, followed by doublet exclusion (FSC-A vs FSC-H), and gating on live cells with a consistent fluorescence profile over time. T cells were identified as $CD20^-$ $CD3^+$, and $CD4^+CD8^-$ cells were further defined as cTFH ($CXCR5^+CD45RA^-$) or Tmem ($CXCR5^-CD45RA^-$). **(b)** Tmem and cTFH populations were phenotyped using memory markers (CD27 vs CCR7) or chemokine receptors (CCR6 vs CXCR3). **(c)** Representative staining of OX-40 and CD25 to identify S-specific responses among non-naïve CD4 T cells following stimulation with hCoV-NL63 spike protein.

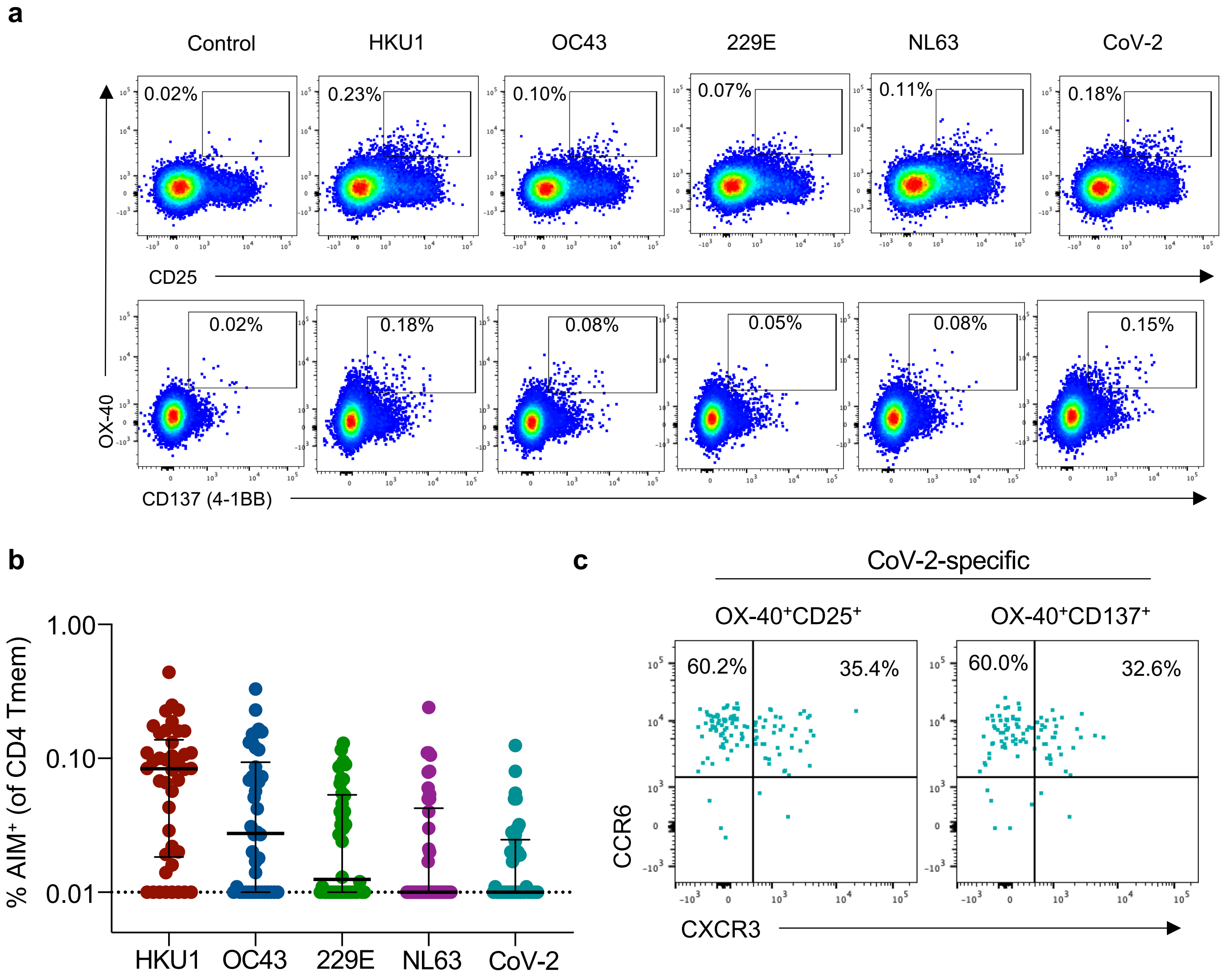

Supplementary figure 2. CD4 Tmem responses measured by different AIM combinations. (a) Comparison of AIM marker readouts (OX-40$^+$CD25$^+$ vs OX-40$^+$CD137$^+$) in a single individual for S-specific responses to all four hCoV antigens and SARS-CoV-2 S. **(b)** Frequency of S-specific Tmem for each antigen (n=42). Bars indicate median. Values represent background subtracted responses; frequencies below 0.01% after background subtraction were assigned a value of 0.01%. Symbols and colours indicate individual donors with SARS-CoV-2 responses >0.01% and are matched to the individuals coded in Figure 2c. **(c)** Comparison of SARS-CoV-2-specific Tmem CCR6 and CXCR3 phenotype based on identification by OX-40$^+$CD25$^+$ or OX-40$^+$CD137$^+$ gates.

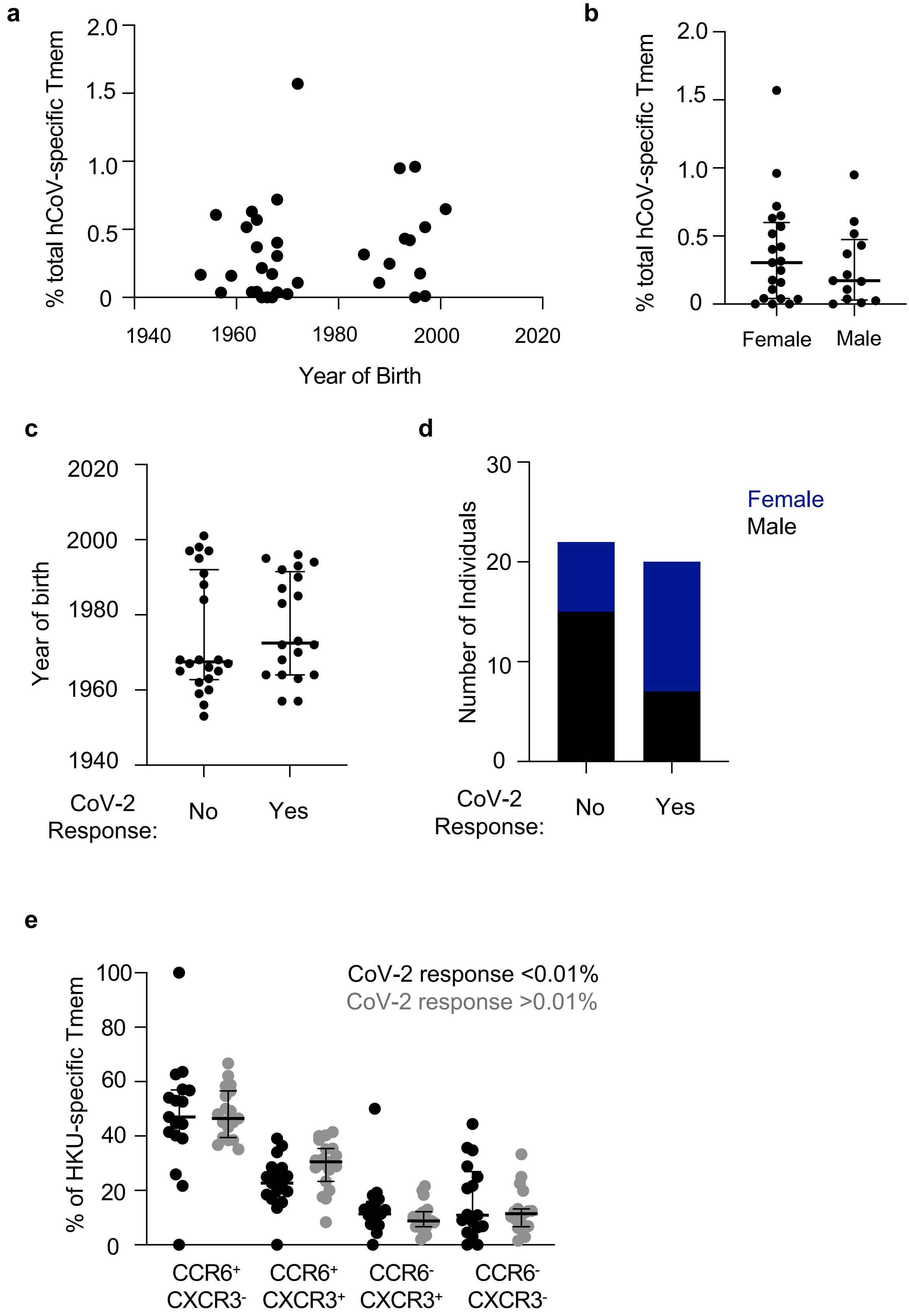

Supplementary figure 3. Demographic and immunological characteristics of total hCoV and CoV-2 cross-reactive responses. (a) Spearman correlation of age and total hCoV Tmem frequency (n=42). **(b)** Total hCoV frequency according to gender (n=42). **(c)** Age and **(d)** gender distribution among individuals with no (<0.01%) CoV-2 S-specific CD4 Tmem responses (n=22) or with CoV-2 responses (n=20). **(e)** Phenotype of HKU1-specific Tmem in individuals without (black, n=17) or with (grey, n=19) cross-reactive CoV-2 responses. Lines and error bars indicate median and interquartile range.

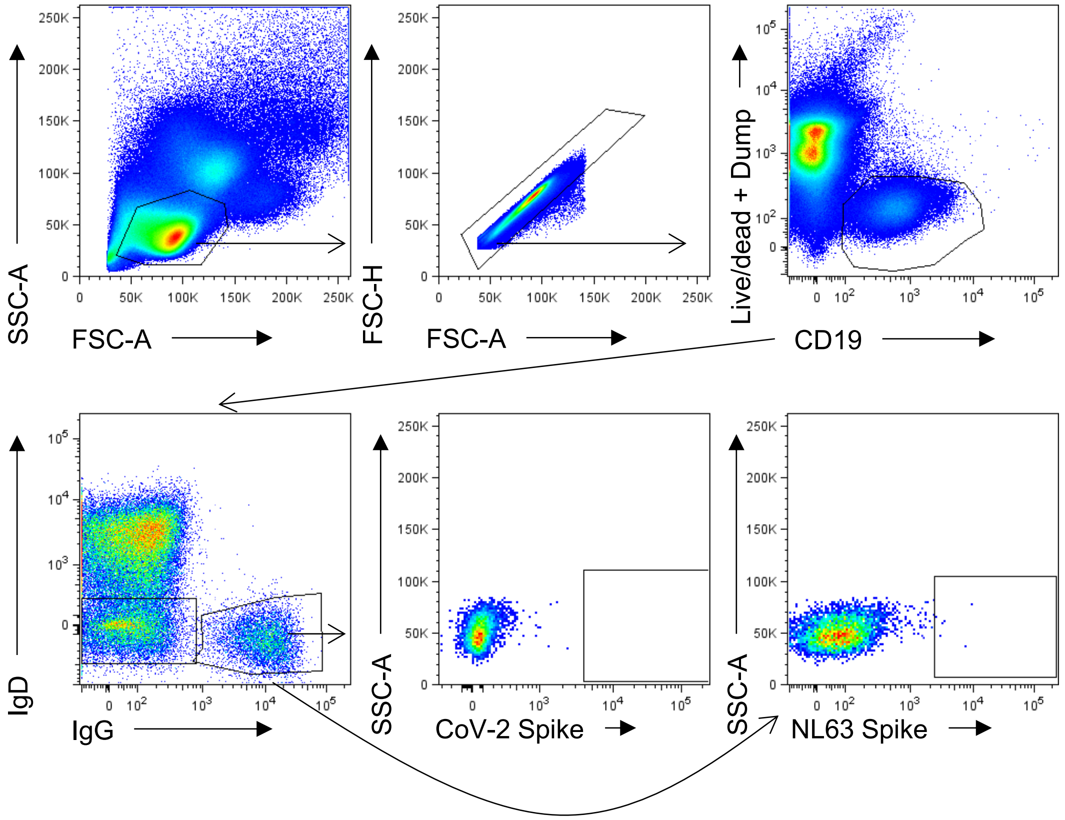

Supplementary figure 4. Gating strategy for resolving antigen-specific B cells
Lymphocytes were identified by FSC-A vs SSC-A gating, followed by doublet exclusion (FSC-A vs FSC-H), and gating on live CD19+ B cells. Class-switched B cells were identified as IgD-, IgG+. Binding to SARS-CoV-2 or NL63 spike was assessed.

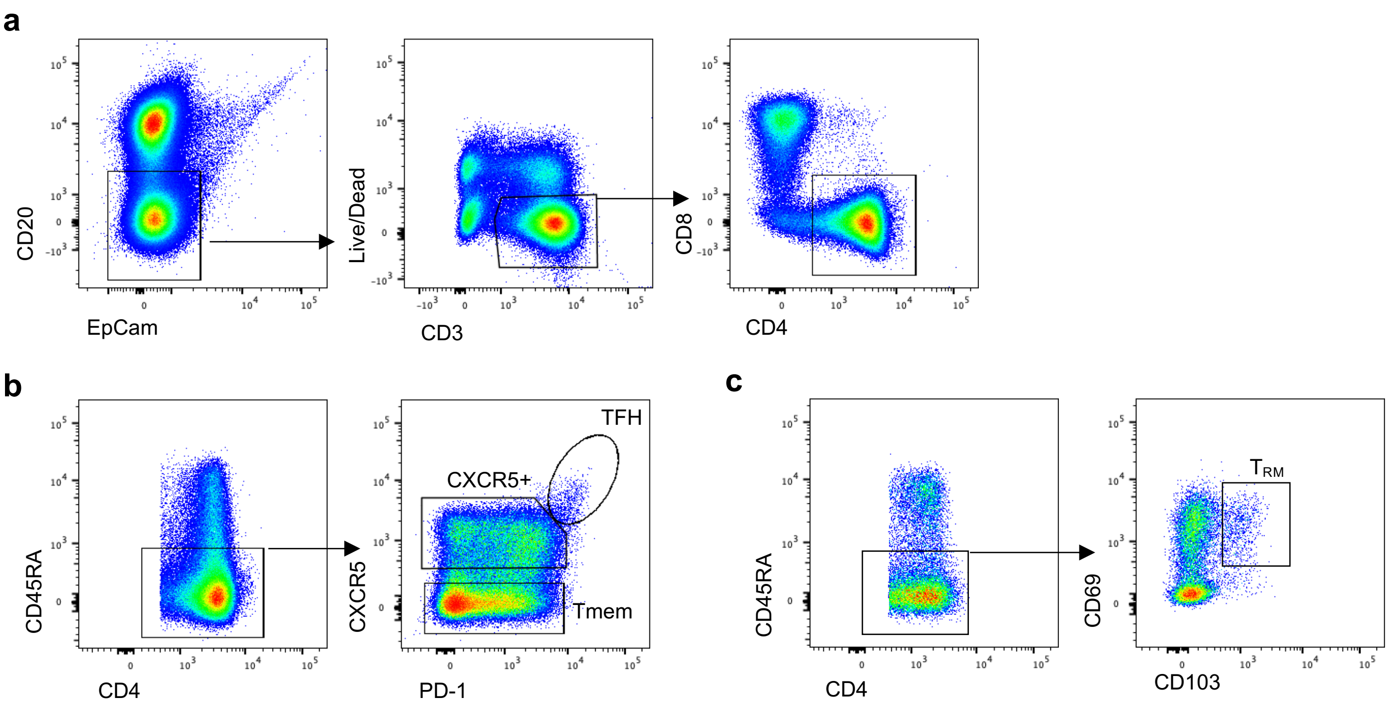

Supplementary figure 5. Lymph node and lung CD4 T cell gating strategy. (a) CD4 T cells in lymph node or lung samples were identified as lymphocytes (identified by forward and side scatter, followed by doublet exclusion) with a CD20-EpCam-Live/Dead-CD3^{+}CD8^{-} CD4^{+} phenotype. **(b)** Memory CD4 T cell subsets in lung draining lymph nodes were identified as CD45RA-, followed by gating based on PD-1 and CXCR5 expression to identify TFH, pre-TFH and Tmem subsets. **(c)** In lung samples, memory CD4 T cells were identified as CD45RA-. T_{RM} cells were identified as CD69^{+}CD103^{+}.

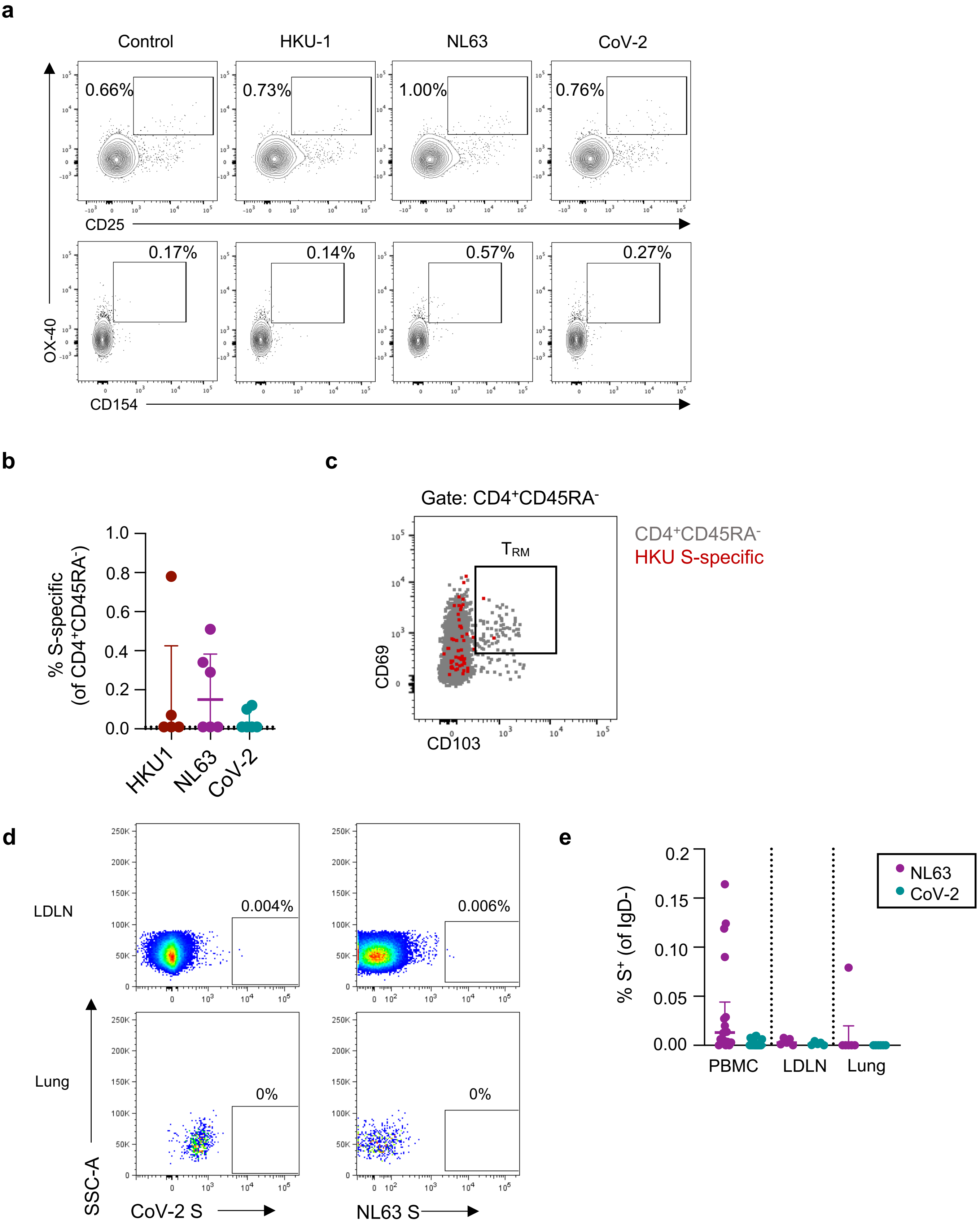

Supplementary figure 6. hCoV and CoV-2 T cell and B cell responses in LDLN and lung. (a) Representative responses to HKU1, NL63 or CoV-2 S antigen stimulation among $CD4^+CD45RA^-$ T cells in the lung. **(b)** Quantification of S-specific T cell responses (n=6 for NL63 and CoV-2, n=5 for HKU1). **(c)** Expression of CD69 and CD103 on total $CD4^+CD45RA^-$ lung T cells (grey) versus HKU S-specific T cells (red). **(d)** Representative staining of NL63 and CoV-2 S-specific MBC in LDLN and lung samples. **(e)** Quantification of S-specific MBC among PBMC samples from the healthy adult cohort (n=18), or human tissue biobank LDLN (n=6) or lung (n=6).
